# Strong magnetic frustration and anti-site disorder causing spin-glass behavior in honeycomb Li_2_RhO_3_

**DOI:** 10.1038/srep14718

**Published:** 2015-10-05

**Authors:** Vamshi M. Katukuri, Satoshi Nishimoto, Ioannis Rousochatzakis, Hermann Stoll, Jeroen van den Brink, Liviu Hozoi

**Affiliations:** 1 Institute for Theoretical Solid State Physics, IFW Dresden, Helmholtzstr. 20, 01069 Dresden, Germany; 2 Institute for Theoretical Chemistry, Universität Stuttgart, Pfaffenwaldring 55, 70550 Stuttgart, Germany

## Abstract

With large spin-orbit coupling, the 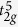 electron configuration in *d*-metal oxides is prone to highly anisotropic exchange interactions and exotic magnetic properties. In 5*d*^5^ iridates, given the existing variety of crystal structures, the magnetic anisotropy can be tuned from antisymmetric to symmetric Kitaev-type, with interaction strengths that outsize the isotropic terms. By many-body electronic-structure calculations we here address the nature of the magnetic exchange and the intriguing spin-glass behavior of Li_2_RhO_3_, a 4*d*^5^ honeycomb oxide. For pristine crystals without Rh-Li site inversion, we predict a dimerized ground state as in the isostructural 5*d*^5^ iridate Li_2_IrO_3_, with triplet spin dimers effectively placed on a frustrated triangular lattice. With Rh-Li anti-site disorder, we explain the observed spin-glass phase as a superposition of different, nearly degenerate symmetry-broken configurations.

For *d*-metal compounds with localized magnetic moments, basic guidelines to soothsay the sign of the nearest-neighbor (NN) magnetic exchange interactions, i. e., the Anderson-Goodenough-Kanamori rules^1^,^2^,^3^, were laid down back in the 1950’s. With one single bridging anion and half-filled *d* states these rules safely predict antiferromagnetic (AF) exchange interactions, as is indeed encountered in numerous magnetic Mott insulators. It is however much harder to anticipate the sign of the couplings for geometries with two bridging ligands and bond angles close to 90°. Illustrative recent examples are the 5*d* honeycomb systems Na_2_IrO_3_ and Li_2_IrO_3_. The signs of the NN Heisenberg *J* and of the additional symmetric Kitaev anisotropy *K* are intensely debated in these iridates, with both *J* < 0, *K* > 0^4^,^5^,^6^ and *J* > 0, *K* < 0^7^,^8^,^9^,^10^,^11^,^12^ sets of parameters being used to explain the available experimental data.

The sizable Kitaev interactions, that is, uniaxial symmetric terms 
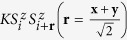
 that cyclically permute on the bonds of a particular hexagonal ring^13^,^14^,^15^, are associated to strong frustration effects and unconventional magnetic ground states displaying, for example, noncollinear order, incommensurability, or spin-liquid behavior^4^,^5^,^6^,^7^,^8^,^11^,^12^,^15^,^16^,^17^. Obviously, in the context of electronic-structure computational methods, such features cannot be thoroughly addressed by periodic total-energy calculations for a given set of predetermined spin configurations. A much more effective strategy is to first focus on individual pairs of NN *d*-metal sites and obtain reliable values for the associated effective magnetic couplings by using *ab initio* many-body quantum chemistry (QC) machinery (for a recent review, see ref. 18). The computed exchange parameters can be subsequently fed to effective spin Hamiltonians to be solved for larger sets of magnetically active lattice sites. Such an approach, earlier, allowed us to establish the signs plus the relative strengths of the Heisenberg and Kitaev interactions in both Na_2_IrO_3_ and Li_2_IrO_3_ and to additionally rationalize the qualitatively different types of AF orders in these two 5*d*^5^ honeycomb iridates^16^,^17^.

The related 4*d*^5^ honeycomb compound Li_2_RhO_3_ is even more puzzling because it features no sign of long-range magnetic order. Instead, an experimental study suggests the presence of a spin-glass ground state^19^. While the spin-orbit couplings (SOC’s) are still sizable for the 4*d* shell and may in principle give rise on the honeycomb lattice to compelling Kitaev physics, to date no conclusive evidence is in this respect available for Li_2_RhO_3_. To shed light on the nature of the essential exchange interactions in Li_2_RhO_3_ we here carry out detailed *ab initio* QC calculations. We show that large trigonal splittings within the Rh *t*_2*g*_ shell, comparable with the strength of the SOC, dismiss a simple picture based on *j*_eff_ = 1/2 and *j*_eff_ = 3/2 effective states^14^,^20^,^21^,^22^. The magnetic properties of the system can still be described, however, in terms of 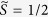 pseudospins. The calculations earmark Li_2_RhO_3_ as a 4*d*-electron system with remarkably large anisotropic magnetic couplings, in particular, FM Kitaev interactions of up to 10–15 meV. The isotropic Heisenberg exchange, on the other hand, features opposite signs on the two sets of structurally distinct links of Rh NN’s. This sign modulation of the NN Heisenberg interactions, with strong ferromagnetic (FM) *J*’s for one type of Rh-Rh bonds and weaker AF couplings for the other pairs of adjacent Rh sites, enables the initial 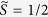 hexagonal network to be mapped onto an effective model of spin-1 dimers on a frustrated triangular lattice. We further address the issue of Rh-Li anti-site disorder in samples of Li_2_RhO_3_. By exact-diagonalization (ED) calculations for an extended spin model that also includes second and third neighbor couplings, we show that the experimentally observed spin-glass behavior can be rationalized as a superposition of different nearly-degenerate symmetry-broken states arising at finite concentration of in-plane spin vacancies.

## Results

### Rh^**4+**^ 4*d* ^5^ electronic structure

The tetravalent rhodium ions in Li_2_RhO_3_ display a 4*d*^5^ valence electron configuration, octahedral ligand coordination and bonding through two bridging ligands. In the simplest picture, i.e., for sufficiently large Rh *t*_2*g*_ − *e*_*g*_ splittings and degenerate *t*_2*g*_ levels, the ground-state electron configuration at each site is a 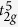 effective *j*_eff_ = 1/2 spin-orbit doublet^14^,^20^,^21^,^22^. For 5*d*^5^ ions in a variety of three-dimensional, layered and chain-like oxides, *ab initio* QC electronic-structure calculations yield excitation energies of 0.6–0.9 eV for the transitions between the *j* ≈ 1/2 and split *j* ≈ 3/2 levels^23^,^24^,^25^,^26^ and indicate values of 0.45–0.5 eV for the strength of the SOC *λ*, in agreement with earlier estimates^27^. Sharp features in the range of 0.6–0.9 eV are indeed found in the resonant x-ray scattering spectra^23^,^24^,^25^,^28^.

The validity of the *j*_eff_ = 1/2 approximation for the ground state of Li_2_RhO_3_ is however questionable since the SOC is substantially weaker for 4*d* elements. Indeed our QC calculations (see Table 1) indicate Rh *t*_2*g*_ splittings *δ* ≈ 0.11 eV, close to values of 0.14–0.16 eV estimated for *λ* in various Rh^4+^ oxides^26^,^29^. For the *ab initio* QC investigation we employed multiconfiguration complete-active-space self-consistent-field (CASSCF) and multireference configuration-interaction (MRCI) calculations^30^, see Supplemental Material (SM) and refs 23,26. With *δ* and *λ* parameters of similar size, the *j*_eff_ = 1/2 and *j*_eff_ = 3/2 states are strongly admixed, as discussed in earlier work^20^,^31^ and illustrated in Table 1. In particular, for the relativistic ground-state wave function the *t*_2*g*_ hole is not equally distributed among the three Rh *t*_2*g*_ levels as for the “true” *j*_eff_ = 1/2 ground state^20^,^21^ but displays predominant *d*_*xy*_ character, close to 60%.

### Magnetic couplings between two adjacent Rh^4+^ ions

Interestingly, while the results for the on-site 4*d*^5^ excitations are quite different as compared to the 5*d*^5^ excitation energies^23^, the computed intersite effective interactions are qualitatively similar to those obtained for the 5*d*^5^ honeycomb iridate Li_2_IrO_3_^17^. The intersite exchange couplings were estimated by MRCI + SOC calculations for embedded fragments having two edge-sharing IrO_6_ octahedra in the active region. As described in earlier work^16^,^17^,^32^ and in SM, the *ab initio* QC data for the lowest four spin-orbit states describing the magnetic spectrum of two NN octahedra is mapped in our scheme onto an effective spin Hamiltonian including both isotropic Heisenberg exchange and symmetric anisotropy. Yet the spin-orbit calculations, CASSCF or MRCI, incorporate all nine triplet and nine singlet states that arise from the two-site 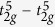 configuration.

**Figure 1:**
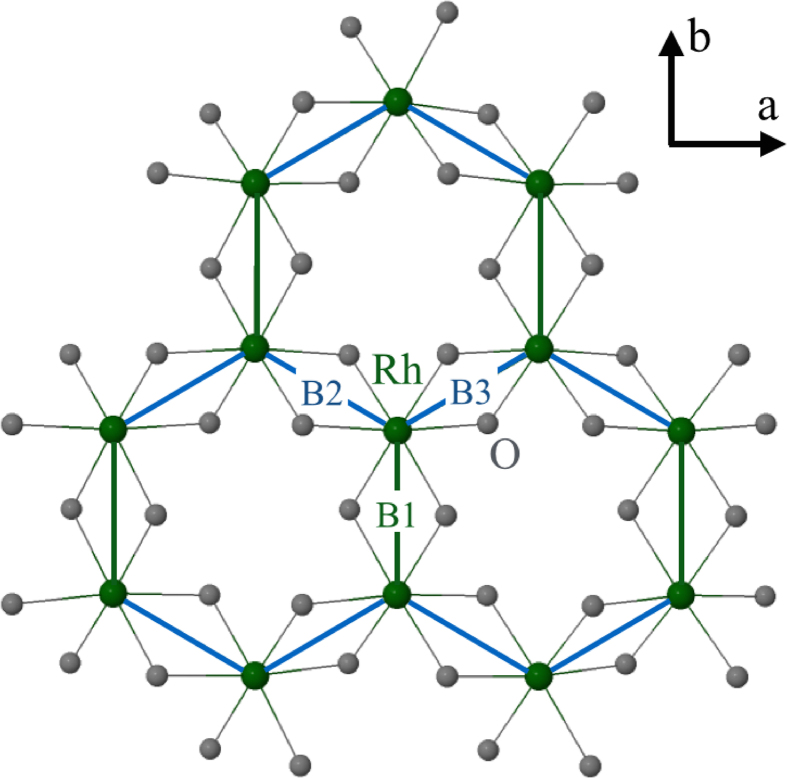
Layered network of edge-sharing RhO_6_ octahedra in Li_2_RhO_3_. The two distinct types, B1 and B2/B3, of NN two-octahedra units and the honeycomb lattice of Rh sites are evidenced.

For on-site Kramers-doublet configurations, the most general symmetry-allowed form of the effective spin Hamiltonian, for a pair of NN ions, is


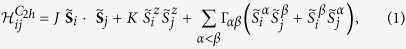


where 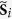, 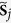 are 1/2 pseudospin operators, *J* is the isotropic Heisenberg interaction, *K* the Kitaev coupling, and the 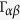 coefficients are off-diagonal elements of the symmetric anisotropic exchange matrix with 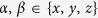. The antisymmetric anisotropic term vanishes since the crystallographic data reported in ref. 33 indicate overall *C*_2 *h*_ point-group symmetry for one block of NN RhO_6_ octahedra, green (B1) bonds in Fig. 1, and only slight deviations from *C*_2 *h*_ for the other type of NN’s, blue (B2 and B3) bonds in Fig. 1. For *C*_2 *h*_ symmetry of the Rh-Rh link, 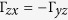. We note that in (1) *α* and *β* stand for components in the *local*, Kitaev bond reference frame. The **z** axis is here perpendicular to the Rh_2_O_2_ plaquette (see SM and refs 14,16,17).

Relative energies for the four low-lying states describing the magnetic spectrum of two NN octahedra and the resulting effective coupling constants are listed in Table 2. For the effective picture of 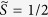 pseudospins assumed in Eq. (1), the set of four eigenfunctions contains the singlet 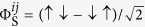 and the triplet components 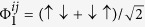, 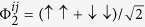, 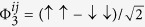. In *C*_2 *h*_ symmetry, the “full” spin-orbit wave functions associated to 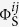, 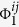, 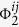 and 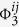 transform according to the *A*_*g*_, *B*_*u*_, *B*_*u*_ and *A*_*u*_ irreducible representations, respectively. Since two of the triplet terms may interact, the most compact way to express the eigenstates of (1) is then 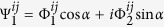, 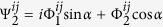, 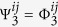 and 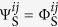. The angle 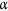 parametrizes the amount of 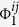

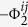
 mixing, related to finite off-diagonal 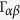 couplings. This degree of admixture is determined by analysis of the full QC spin-orbit wave functions. The effective parameters provided in Table 2 are obtained for each type of Rh-Rh link by using the *E*_1_, *E*_2_, *E*_3_, *E*_S_ MRCI relative energies and the 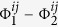 mixing coefficients (see SM).

**Figure 2:**
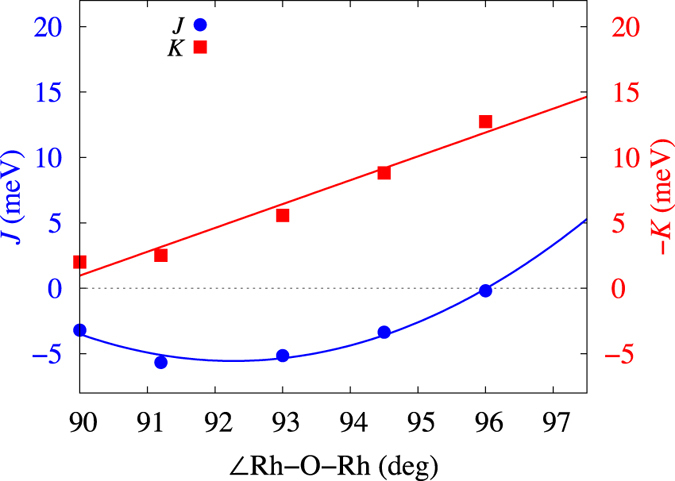
Dependence of the NN *J* and *K* on the Rh-O-Rh bond angle for an idealized structural model where all Rh-O bond lengths are set to the average value in the experimental crystal structure^33^. MRCI+SOC results are shown. The variation of the Rh-O-Rh angles is the result of gradual trigonal compression of the O octahedra.

For B1 links, we find that both *J* and *K* are FM. While by MRCI calculations *K* always comes FM in spin-orbit coupled honeycomb systems^16^,^17^, the FM *J* for the B1 bonds has much to do with the peculiar kind of dependence on the amount of trigonal squashing of the oxygen octahedra and consequently on the variation of the Rh-O-Rh angles of the Rh_2_O_2_ plaquette. The latter increase to values larger than 90° for finite trigonal compression. This dependence of the NN *J* on the Rh-O-Rh bond angles is illustrated in Fig. 2 for a simplified structural model of Li_2_RhO_3_ where the Rh-O bond lengths are all the same, set to the average bond length in the experimental crystal structure^33^. It is seen that *J* displays a parabolic behavior, with a minimum of about −5 meV in the interval 92–93° and a change of sign to AF couplings around 96°. For the B1 Rh-Rh links, the Rh-O-Rh bond angle is 93.4°, close to the value that defines the minimum in Fig. 2. The difference between the ≈ − 5 meV minimum of Fig. 2 and the ≈ − 10 meV result listed in Table 2 comes from additional distortions of the O octahedra in the actual structure (see the footnotes in Table 2 and ref. 33), not included in the idealized model considered for the plot in Fig. 2. An even stronger FM *J* was computed for the B1 type bonds in the related compound Li_2_IrO_3_^17^. In Na_2_IrO_3_, on the other hand, the Ir-O-Ir bond angles are >97° and the NN *J* turns AF on all short Ir-Ir links^16^.

For the B2 and B3 links, we derive a FM Kitaev term and an AF Heisenberg interaction, again qualitatively similar to the QC data for Li_2_IrO_3_^17^. We assign the AF value of the NN *J* on the B2/B3 units to the slightly larger Rh-O-Rh bond angle, which as shown in Fig. 2 pulls the *J* towards a positive value, and most importantly to additional distortions that shift the bridging ligands on the Rh-O_2_-Rh B2/B3 plaquettes in opposite senses parallel to the Rh-Rh axis^33^. The role of these additional distortions on the B2/B3 units was analyzed in detail in ref. 16 and shown to enhance as well the AF component to the intersite exchange.

### Effect of longer-range exchange interactions and occurence of spin-glass ground state

**Figure 3:**
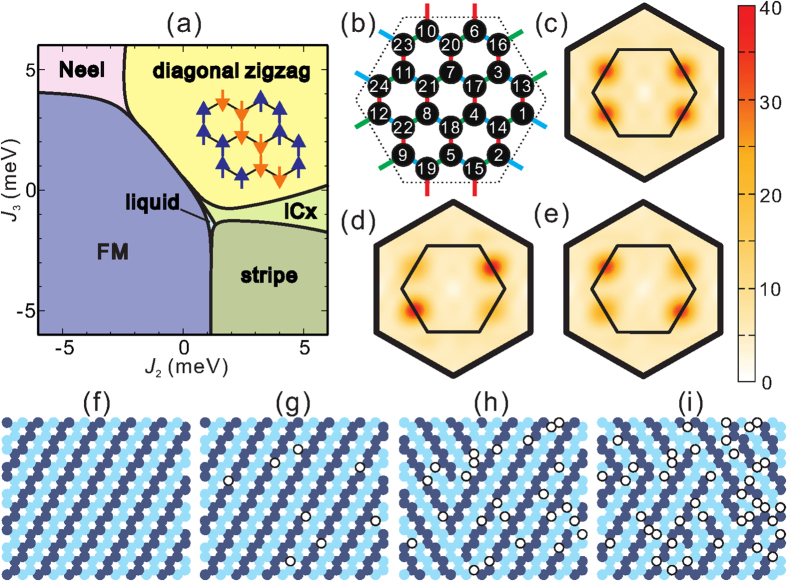
(**a**) Phase diagram for the effective model of Eq. (1) supplemented by 2nd- and 3rd-neighbor isotropic couplings *J*_2_, *J*_3_. The NN effective interaction constants are set to the QC values provided in Table 2. The spin structure for the diagonal zigzag state is also shown. (**b**) Sketch of the cluster used in the ED calculations; the site index *p* runs from 1 to 24. Spin structure factors for *J*_2_ = *J*_3_ = 3 with either (**c**) no spin defects or two spin defects at (**d**) (*p*_1_, *p*_2_) = (17, 20) and (**e**) (*p*_1_, *p*_2_) = (17, 18). Spin configurations for the simplified Ising model are shown for spin-defect concentrations of (**f**) *x* = 0, (**g**) *x* ~ 2%, (**h**) *x* ~ 7%, (**i**) *x* ~ 12%. Filled dark and light circles indicate opposite spin directions. Open circles show the position of spin defects.

For further insights into the magnetic properties of Li_2_RhO_3_, we carried out ED calculations for an extended spin Hamiltonian that in addition to the NN terms of Eq. (1) incorporates longer-range second- and third-neighbor Heisenberg interactions *J*_2_ and *J*_3_^5^,^7^,^8^,^9^. We used clusters of 24 sites with periodic boundary conditions^4^,^16^,^17^ and the quantum chemically derived NN coupling constants listed in Table 2. The static spin-structure factor 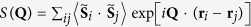 was calculated as function of variable *J*_2_ and *J*_3_ parameters. For a given set of *J*_2_ and *J*_3_ values, the dominant order is determined according to the wave number **Q **= **Q**_*max*_ providing a maximum value of *S*(**Q**). The resulting phase diagram is shown in Fig. 3(a). Given the similar structure of the NN magnetic interactions, it is somewhat similar to that obtained in our previous study on Li_2_IrO_3_^17^. Six different regions can be identified for 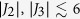 meV: FM, Néel, Kitaev spin liquid, stripy, diagonal zigzag and incommensurate **Q** (ICx) phases. The Kitaev spin liquid, stripy and incommensurate phases in strongly spin-orbit coupled honeycomb 5*d*^5^ systems were analyzed in a number of earlier studies^4^,^6^,^7^,^8^,^16^. The detailed nature of the diagonal zigzag and incommensurate ICx ground states for large FM *J* on one set of NN links was described in ref. 17. Remarkably, for *J*(B1) much larger than *K*(B1) and *J*(B2), the initial hexagonal 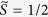 lattice can be mapped onto an effective triangular model of *triplet* dimers on the B1 bonds^17^.

Since *J*_2_ and *J*_3_ are expected to be AF in honeycomb *d*^5^ oxides^8^,^9^, the most likely candidate for the magnetic ground state of “clean” crystals of Li_2_RhO_3_, according to our results, is the diagonal zigzag state (see Fig. 3) and is found to be stable in a wide region of 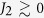 and 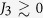. Experimentally, however, a spin-glass ground state was determined, with a spin freezing temperature of ~6 K^19^. As possible cause of the observed spin-glass behavior in Li_2_RhO_3_ we here investigate the role of Li-Rh site intercalation. Significant disorder on the cation sublattice is a well known feature in Li_2_*M*O_3_ compounds. A typical value for the degree of Li^+^ −*M*^4+^ site inversion in these materials is 10–15%^34^,^35^. Partial substitution of the “in-plane” Rh^4+^ ions by nonmagnetic Li^+^ species introduces spin defects in the 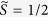 honeycomb layer. On the 24-site cluster employed for our ED calculations, 10–15% site inversion translates in replacing two 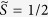 centers by vacancies. Hereafter, we denote the two spin defects as *p*_1_ and *p*_2_.

The effect of spin vacancies on the static spin-structure factor in the diagonal zigzag phase (*J*_2_ = *J*_2_ = 3) is shown in Fig. 3(d,e). For comparison, the static spin-structure factor is also plotted in Fig. 3(c) for the ideal case without spin defects. In the absence of “defects”, the ground state is characterized in the bulk limit by symmetry-broken long-range order with either 
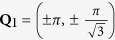
 or 
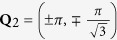
 wave vectors. Since the two symmetry-breaking states are degenerate, the structure factor displays four peaks, at **Q** = **Q**_1_ and **Q** = **Q**_2_ [see Fig. 3(c)]. However, by introducing spin vacancies, the degeneracy may be lifted via impurity pinning effects. For example, when the two defects occupy positions 17 and 20 [(*p*_1_, *p*_2_) = (17, 20), see Fig. 3(b)] the spin structure defines one of the symmetry-breaking states with **Q** = **Q**_1_ [Fig. 3(d)]; likewise, defects at (*p*_1_, *p*_2_) = (17, 18) yield a state with **Q** = **Q**_2_ [Fig. 3(e)]. In other words, two different kinds of dominant short-range order can be obtained with anti-site disorder. The “locally” favored symmetry-breaking direction depends on the relative positions of the spin vacancies. In a macroscopic system, such “local” domains displaying different symmetry-breaking ordering directions are randomly distributed. Additional *frustration* is expected to arise because it is not possible to match two differently ordered domains without an emerging “string”. It is therefore likely that by creating some amount of spin defects the long-range zigzag order disappears and the resulting state is perceived as a spin glass at low temperature. A similar mechanism was proposed for the isotropic Heisenberg-Kitaev and *J*_1_-*J*_2_-*J*_3_ models^5^.

An early well known example of frustration induced through the competition between two different, degenerate spin configurations is the two-dimensional Ising model on a square lattice with randomly distributed, competing FM and AF bonds^36^. To investigate how the diagonal zigzag state is destroyed by increasing the concentration of spin defects, we also studied a simplified Ising model with *J* = − ∞ for the B1 bonds, *J*_2_ = *J*_3_ and all other interactions set to zero. This is a reasonable approximation for the honeycomb layers of Li_2_RhO_3_ since the diagonal zigzag phase essentially consists of alternating spin-up and spin-down chains [see sketch in Fig. 3(a)]. Spin structures obtained this way for various spin-defect concentrations *x* are shown in Fig. 3(f–i). For *x* = 0 the symmetry-breaking diagonal zigzag state is realized, with degenerate **Q** = **Q**_1_ and **Q** = **Q**_2_ spin structures. At finite, low concentration *x* ~ 2% those two configurations are no longer degenerate since one of them features slightly lower ground-state energy. We still have in this case a “macroscopically stable” ground state. At intermediate defect concentration *x* ~ 7% the long-range order is in a strict sense destroyed. However, the large domain walls with either **Q** = **Q**_1_ and **Q** = **Q**_2_ seem to survive. At higher concentration *x* ~ 12% the long-range order disappears completely. Moreover, we can now identify a mixture of local structures with different symmetry-breaking directions [see Fig. 3(i)].

## Discussion

In sum, we have calculated the microscopic neareast-neighbor magnetic interactions between effective 1/2 spins in Li_2_RhO_3_ and uncovered a substantial difference between the two types of bonds that are present: one is dominated by Heisenberg and the other by Kitaev types of couplings. The latter give rise to strong frustration, even if the interactions are predominantly ferromagnetic. In this setting we have additionally considered the effect of the presence of anti-site disorder. Experimentally the in-plane spin-defect concentration in Li_2_RhO_3_ has been estimated as *x* = 10–15%^34^,^35^. Based on our theoretical findings it is likely that the observed spin-glass behavior arises from the combination of such anti-site disorder and strongly frustrating magnetic interactions, in particular, the different Kitaev/Heisenberg dominated magnetic bonds and the Ising-like physics associated with the triplet dimer formation that results from there.

Our combined *ab initio* and effective-model calculations on both Li_2_RhO_3_ and related *d*^5^ honeycomb iridates^16^,^17^,^23^ indicate that a description in terms of on-site 1/2 pseudospins can well account for the diverse magnetic properties of these systems. While alternative models rely on the formation of delocalized, quasimolecular orbitals^37^,^38^ and for Li_2_RhO_3_ downplay the role of spin-orbit interactions^38^, here we show that the latter give rise in Li_2_RhO_3_ to anisotropic Kitaev interactions the same magnitude as in 5*d* iridates^16^,^17^,^39^. That happens in spite of having a Rh *t*_2*g*_ splitting *δ* and a spin-orbit coupling *λ* of similar magnitude, the same way similar sets of Ir *δ* and *λ* parameters in CaIrO_3_^40^ still generate symmetric anisotropic exchange terms in the range of 10 meV (work is in progress).

## Methods

The Molpro QC package was employed for all *ab initio* calculations^41^. To analyze the electronic ground state and the nature of the *d*-*d* excitations, a cluster consisting of one reference RhO_6_ octahedron plus three NN RhO_6_ octahedra and 15 nearby Li ions was used. The magnetic spectrum for two Rh^4+^ ions was obtained from calculations on a cluster containing two reference and four NN RhO_6_ octahedra plus the surrounding 22 Li ions, see SM for details. The farther solid-state environment was in both cases modeled as a finite array of point charges fitted to reproduce the crystal Madelung field in the cluster region. The spin-orbit treatment was carried out according to the procedure described in ref. 42, using spin-orbit pseudopotentials for Rh.

## Additional Information

**How to cite this article**: Katukuri, V. M. *et al.* Strong magnetic frustration and anti-site disorder causing spin-glass behavior in honeycomb Li_2_RhO_3_. *Sci. Rep.* **5**, 14718; doi: 10.1038/srep14718 (2015).

## Supplementary Material

Supplementary Information

## Figures and Tables

**Table 1:** Rh^4**+**^ 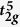 states in Li_2_RhO_3_, with composition of the wave functions (hole picture) and relative energies (meV).

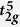 **states**	**Relative energies**	**Wave-function composition (normalized weights)**
CASSCF:		
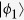	0	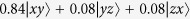
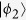	107	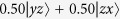
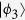	110	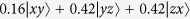
CASSCF+SOC:		
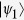	0	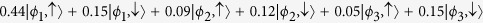
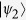	210	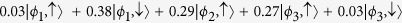
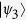	265	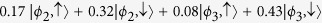

CASSCF results without and with SOC are shown. Only the three Rh *t*_2*g*_ orbitals were active^30^ in CASSCF. By subsequent MRCI calculations, the relative energies of these states change to 0, 85, 95 without SOC and 0, 235, 285 meV with SOC included. Only one component of the Kramers’ doublet is shown for each spin-orbit wave function.

**Table 2:** Relative energies of the four low-lying magnetic states and the associated effective exchange couplings (meV) for two NN RhO_6_ octahedra in Li_2_RhO_3_.

**Energies & effective couplings**	**B1**^1^	**B2/B3**^2^
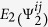	0.0	0.0
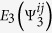	2.5	−3.3
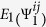	4.5	4.6
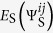	13.5	1.9
*J*	−10.2	2.4
*K*	−2.9	−11.7
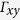	−1.3	3.6
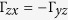	2.8	1.6

Two distinct types of such [Rh_2_O_10_] units, B1 and B2/B3 (see text), are found experimentally^33^. Results of spin-orbit MRCI calculations are shown, with a *local* coordinate frame for each Rh-Rh link (*x* along the Rh-Rh bond, *z* perpendicular to the Rh_2_O_2_ plaquette). The form of the actual lattice spin model is detailed in the SM.

^1^
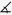
(Rh-O-Rh)=93.4°, *d*(Rh-Rh)=2.95 (×2), *d*(Rh-O_1,2_)=2.03 Å.

^2^
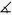
(Rh-O-Rh)=94.1°, *d*(Rh-Rh)=2.95 (×4), *d*(Rh-O_1_)=2.03, *d*(Rh-O_2_)=2.00 Å. O_1_ and O_2_ are the two bridging O’s.
