# Supplementary material for: Strong magnetic frustration and anti-site disorder causing spin-glass behavior in honeycomb Li2RhO3
Source: Sci Rep. 2015 Oct 5;5:14718. doi: 10.1038/srep14718 (PMC4593009; doi:10.1038/srep14718)
Supplement: Supplementary Information [file srep14718-s1.pdf]

Supplementary Information — Strong magnetic frustration and anti-site disorder causing spin-glass behavior in honeycomb Li_2RhO_3

Vamshi M. Katukuri[1,*], Satoshi Nishimoto[1,+], Ioannis Rousochatzakis[1], Hermann Stoll[2], Jeroen van den Brink[1], and Liviu Hozoi[1]

[1]Institute for Theoretical Solid State Physics, IFW Dresden, Helmholtzstr. 20, 01069 Dresden, Germany
[2]Institute for Theoretical Chemistry, Universität Stuttgart, Pfaffenwaldring 55, 70550 Stuttgart, Germany
*v.m.katukuri@ifw-dresden.de
+s.nishimoto@ifw-dresden.de

1 Quantum chemistry calculations

Rh^{4+} $4d^5$ electronic structure. To analyze the ground state and the nature of the valence *d*-*d* excitations in Li_2RhO_3, we performed *ab initio* quantum chemistry (QC) calculations on a cluster consisting of one reference RhO_6 octahedron plus three nearest-neighbor (NN) RhO_6 octahedra and 15 nearby Li ions. The surrounding solid-state matrix was modeled as a finite array of point charges fitted to reproduce the crystal Madelung field in the cluster region. We used energy-consistent relativistic pseudopotentials for Rh, with quadruple-zeta basis sets for the valence shells of the reference Rh ion and triple-zeta basis functions for the Rh NN's.[1] The oxygen ligands of the central octahedron were represented by all-electron triple-zeta basis sets.[2] We also applied two Rh *f* polarization functions[1,2] for the central Rh site. For the Li^+ NN's we employed total-ion effective potentials and a single *s* valence basis function.[3] To obtain a clear picture on the effect of spin-orbit coupling (SOC) at the central Rh^{4+} site and to keep the analysis of the spin-orbit coupled wave functions tractable, we cut off the magnetic couplings with the adjacent Rh^{4+} ions by modeling the latter as closed-shell Pd^{4+} t_{2g}^6 species. Such a procedure is frequently employed in QC studies on transition-metal systems, see, e.g., Refs. 4–6.

All computations were performed with the MOLPRO QC pacakge.[7] In the complete-active-space self-consistent-field (CASSCF) calculations, only the $4d$ t_{2g} orbitals at the central Rh site and five electrons were included in the active space. All O $2p$ and Rh t_{2g} electrons at the central octahedron were correlated in the subsequent multireference configuration interaction (MRCI) calculations.

Magnetic couplings between two adjacent Rh^{4+} ions. The magnetic spectrum of two NN Rh^{4+} ions was obtained from CASSCF and MRCI spin-orbit calculations on units of two edge-sharing RhO_6 octahedra. To accurately describe the charge distribution at sites in the immediate neighborhood, we also included in the actual cluster the closest 22 Li^+ ions and the four adjacent RhO_6 octahedra around the reference $[Rh_2O_{10}]$ fragment. As for the single-site calculations, we used energy-consistent relativistic pseudopotentials with quadruple-zeta basis sets for the valence shells of the two reference Rh ions,[1] all-electron quintuple-zeta basis sets for the two bridging ligands[2] and triple-zeta basis functions for the other O's of the two reference octahedra.[2] Additionally, we employed two Rh *f*[1] and four O *d* polarization functions for the two central Rh ions and the two bridging ligands,[2] respectively. The NN Rh^{4+} sites were once again modeled as closed-shell Pd^{4+} t_{2g}^6 species.

Multiconfiguration reference wave functions were first generated by CASSCF calculations. For two NN RhO_6 octahedra, the finite set of Slater determinants was defined in the CASSCF treatment in terms of ten electrons (2×5) and six Rh t_{2g} orbitals (2×3). The SCF optimization was carried out for an average of the lowest nine singlet and the nine triplet states associated with this manifold. All these 18 states entered the spin-orbit calculations, both at the CASSCF and MRCI levels. On top of the CASSCF reference, the MRCI expansion additionally includes single and double excitations from the Rh t_{2g} shells and the $2p$ orbitals of the bridging ligands. A similar strategy of explicitly dealing only with selected groups of ligand orbitals was earlier adopted in QC studies on both $3d$[8–11] and $5d$[4–6,12,13] compounds, with results in good agreement with the experiment.[4,6,8–12] To separate the Rh $4d$ and O $2p$ valence orbitals into different groups, we used the Pipek-Mezey[14] orbital localization module available in MOLPRO.[7]

2 Effective spin Hamiltonian

The effective magnetic Hamiltonian for two adjacent Rh sites is most conveniently written in a local reference frame $\{\mathbf{X},\mathbf{Y},\mathbf{Z}\}$ with the Rh-Rh bond along the $\mathbf{X}$ axis and $\mathbf{Z}$ perpendicular to the Rh_2O_2 plaquette. For C_{2h} point-group symmetry, it reads

$$\mathscr{H}_{\langle ij\rangle} = \tilde{\mathbf{S}}_i \cdot \begin{pmatrix} J^{(0)}+A & 0 & 0 \\ 0 & J^{(0)}+B & C \\ 0 & C & J^{(0)}-(A+B) \end{pmatrix} \cdot \tilde{\mathbf{S}}_j\,. \tag{1}$$

Due to the inversion center, the antisymmetric anisotropy does not show up here. Further, for the symmetric anisotropic term, only one offdiagonal element is nonzero in C_{2h}.

A straightforward diagonalization of $\mathscr{H}_{\langle ij\rangle}$ yields the following eigenvalues and eigenfunctions:

$$\begin{aligned} E_S &= -\frac{3J^{(0)}}{4}, & \Phi_S &= \frac{\uparrow\downarrow-\downarrow\uparrow}{\sqrt{2}}, \\ E_1 &= \frac{J^{(0)}+A+\sqrt{(A+2B)^2+4C^2}}{4}, & \Phi_1 &= \frac{\uparrow\downarrow+\downarrow\uparrow}{\sqrt{2}}, \\ E_2 &= \frac{J^{(0)}+A-\sqrt{(A+2B)^2+4C^2}}{4}, & \Phi_2 &= \frac{\uparrow\uparrow+\downarrow\downarrow}{\sqrt{2}}, \\ E_3 &= \frac{J^{(0)}-2A}{4}, & \Phi_3 &= \frac{\uparrow\uparrow-\downarrow\downarrow}{\sqrt{2}}. \end{aligned} \tag{2}$$

Here Φ_S is the total spin singlet and Φ_{1-3} are combinations of the three triplet components. The latter are degenerate in the plain Heisenberg model.

The above diagonalization procedure is equivalent with a rotation of the coordinate system $\{\mathbf{X},\mathbf{Y},\mathbf{Z}\}$ around $\mathbf{X}$ by an angle α to a new frame $\{\mathbf{X}',\mathbf{Y}',\mathbf{Z}'\}$ in which the symmetric anisotropic exchange matrix is diagonal.[15] $\{\mathbf{X}',\mathbf{Y}',\mathbf{Z}'\}$ are also referred to as principal axes and the angle α is given by

$$\tan(2\alpha) = \frac{2C}{A+2B}\,. \tag{3}$$

In C_{2h} symmetry, the Φ_S, Φ_1, Φ_2 and Φ_3 spin-orbit wave functions transform according to the A_g, B_u, B_u and A_u irreducible representations, respectively. Since states Φ_1 and Φ_2 belong to the same irreducible representation B_u, they are in general

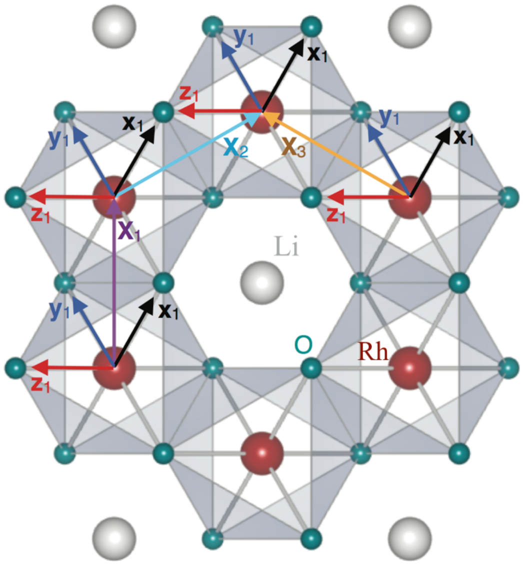

Figure 1. Local reference frames $\{\mathbf{X}_b,\mathbf{Y}_b,\mathbf{Z}_b\}$ and $\{\mathbf{x}_b,\mathbf{y}_b,\mathbf{z}_b\}$ introduced for each of the three different types of NN Rh-Rh links.

admixed, i.e., in the reference frame $\{\mathbf{X},\mathbf{Y},\mathbf{Z}\}$ the corresponding eigenfunctions should be written as

$$\Psi_1 = \Phi_1 \cos\alpha + i\Phi_2 \sin\alpha\,,$$
$$\Psi_2 = i\Phi_1 \sin\alpha + \Phi_2 \cos\alpha\,. \tag{4}$$

The mixing parameter $\xi = \sin\alpha$ is given by

$$i\xi = \langle\Phi_2|\Psi_1\rangle = \langle\Phi_1|\Psi_2\rangle \tag{5}$$

and is explicitly obtained from the QC data.

For Rh-Rh links along the crystallographic b axis (labeled B1) the symmetry of a block of two edge-sharing octahedra is C_{2h} while for the other Rh-Rh links (labeled B2/B3) the Rh-O bonds on the Rh-O_2-Rh plaquette have different lengths and the symmetry is lowered to C_i.[16] Since the QC calculations were actually performed in C_1 symmetry, to determine the nature of each of the lowest four spin-orbit states, we explicitly computed the dipole and quadrupole transition matrix elements within that manifold. Standard selection rules and the nonzero dipole and quadrupole matrix elements in the QC outputs then clearly indicate which state is which. We also carried out the transformation of the spin-orbit wave functions from the usual $\{L_1,M_{L_1},L_2,M_{L_2},S,M_S\}$ basis in standard QC programs to the $\{\tilde{S}_1,\tilde{S}_2,\tilde{M}_{S_1},\tilde{M}_{S_2}\}$ basis. This allows the study of Φ_1–Φ_2 mixing due to the offdiagonal Γ_{yz} and Γ_{zx} couplings. With such an analysis, we find that in Li_2RhO_3 the weight of Φ_1 in Ψ_1 (and of Φ_2 in Ψ_2) is 73% for links B1 and 94% for B2/B3.

Using Eqs. (2), (3) and (5) we obtain the effective coupling parameters of (1) as:

$$\begin{aligned}
J^{(0)} &= \frac{1}{3}(E_1+E_2+E_3)-E_S,\\
A &= \frac{2}{3}(E_1+E_2)-\frac{4}{3}E_3,\\
B &= \frac{1}{2}\left[-A\pm\frac{2(E_1-E_2)}{\sqrt{1+\eta^2}}\right],\ \text{with } \eta=\frac{2\xi\sqrt{1-\xi^2}}{1-2\xi^2},\\
C &= \frac{\eta(A+2B)}{2}.
\end{aligned} \tag{6}$$

In the local Kitaev reference system $\{\mathbf{x},\mathbf{y},\mathbf{z}\}$, that is rotated from the reference frame $\{\mathbf{X},\mathbf{Y},\mathbf{Z}\}$ by 45° about the $\mathbf{Z}=\mathbf{z}$ axis (see Fig.1 and Refs. 5, 17), the Hamiltonian given in expression (1) above is transformed into equation (1) of the main text. For the latter, the exchange interaction parameters are given by :[5]

$$J = J^{(0)}+\frac{A+B}{2}\ ,\ \ K=-\frac{3}{2}(A+B)\ ,\ \ \Gamma_{xy}=\frac{A-B}{2}\equiv D\ ,\ \ \Gamma_{zx}=\frac{-C}{\sqrt{2}}\ ,\ \ \Gamma_{yz}=\frac{C}{\sqrt{2}}\,. \tag{7}$$

3 Lattice spin model, notations and *ab initio* effective couplings

For each type of Rh-Rh link, we used two different local axes frames, namely $\{\mathbf{x}_b,\mathbf{y}_b,\mathbf{z}_b\}$ and $\{\mathbf{X}_b,\mathbf{Y}_b,\mathbf{Z}_b\}=\{\frac{\mathbf{x}_b+\mathbf{y}_b}{\sqrt{2}},\frac{-\mathbf{x}_b+\mathbf{y}_b}{\sqrt{2}},\mathbf{z}_b\}$, where $b\in\{1,2,3\}$ labels the type of bond.[5] Let us choose our *global* frame to be $\{\mathbf{x},\mathbf{y},\mathbf{z}\}=\{\mathbf{x}_1,\mathbf{y}_1,\mathbf{z}_1\}$. The relation between the local axes $\{\mathbf{X}_b,\mathbf{Y}_b,\mathbf{Z}_b\}$ and the global frame is then the following (see Fig. 1):

$$\{\mathbf{X}_1,\mathbf{Y}_1,\mathbf{Z}_1\}=\{\frac{\mathbf{x}+\mathbf{y}}{\sqrt{2}},\frac{-\mathbf{x}+\mathbf{y}}{\sqrt{2}},\mathbf{z}\},\ \ \{\mathbf{X}_2,\mathbf{Y}_2,\mathbf{Z}_2\}=\{\frac{\mathbf{x}-\mathbf{z}}{\sqrt{2}},-\frac{\mathbf{x}+\mathbf{z}}{\sqrt{2}},\mathbf{y}\},\ \ \{\mathbf{X}_3,\mathbf{Y}_3,\mathbf{Z}_3\}=\{\frac{\mathbf{y}+\mathbf{z}}{\sqrt{2}},\frac{-\mathbf{y}+\mathbf{z}}{\sqrt{2}},\mathbf{x}\}. \tag{8}$$

We note that the system is invariant under two-fold rotations around the vector $\mathbf{X}_1$. This is why we must choose $\mathbf{X}_3$ to be the rotated version of $\mathbf{X}_2$, i.e., $\mathbf{C}_2\cdot\mathbf{X}_2=\mathbf{X}_3$. If we choose the opposite direction for $\mathbf{X}_3$, then we must change the sign of the coupling C.

Let us now write down the NN terms of the Hamiltonian for the three types of bonds $b=1,2,3$. In the reference frame $\{\mathbf{X}_b,\mathbf{Y}_b,\mathbf{Z}_b\}$,

$$\begin{aligned}
\mathscr{H}_{\langle ij\rangle\in b} &= J_b^{(0)}\mathbf{S}_i\cdot\mathbf{S}_j+A_bS_i^{X_b}S_j^{X_b}+B_bS_i^{Y_b}S_j^{Y_b}-(A_b+B_b)S_i^{Z_b}S_j^{Z_b}+C_b(S_i^{Y_b}S_j^{Z_b}+S_i^{Z_b}S_j^{Y_b})\\
&= J_b\mathbf{S}_i\cdot\mathbf{S}_j+K_bS_i^{Z_b}S_j^{Z_b}+D_b(S_i^{X_b}S_j^{X_b}-S_i^{Y_b}S_j^{Y_b})+C_b(S_i^{Y_b}S_j^{Z_b}+S_i^{Z_b}S_j^{Y_b})\ ,
\end{aligned} \tag{9}$$

where $J_b = J_b^{(0)} + \frac{1}{2}(A_b + B_b)$, $K_b = -\frac{3}{2}(A_b + B_b)$ and $D_b = \frac{1}{2}(A_b - B_b)$. For simplicity, we replace in the following $J_{b=1}$ by J, $J_{b=2,3}$ by J' and similarly for the remaining parameters. Eqs. (9) and (8) then yield

$$\mathscr{H}_{\langle ij\rangle\in b=1} = J\mathbf{S}_i\cdot\mathbf{S}_j + KS_i^zS_j^z + D(S_i^xS_j^y + S_i^yS_j^x) + \frac{C}{\sqrt{2}}(S_i^yS_j^z + S_i^zS_j^y - S_i^xS_j^z - S_i^zS_j^x)\,,$$

$$\mathscr{H}_{\langle ij\rangle\in b=2} = J'\mathbf{S}_i\cdot\mathbf{S}_j + K'S_i^yS_j^y + D'(-S_i^xS_j^z - S_i^zS_j^x) + \frac{C'}{\sqrt{2}}(-S_i^xS_j^y - S_i^yS_j^x - S_i^zS_j^y - S_i^yS_j^z)\,,$$

$$\mathscr{H}_{\langle ij\rangle\in b=3} = J'\mathbf{S}_i\cdot\mathbf{S}_j + K'S_i^xS_j^x + D'(S_i^yS_j^z + S_i^zS_j^y) + \frac{C'}{\sqrt{2}}(-S_i^xS_j^y - S_i^yS_j^x + S_i^xS_j^z + S_i^zS_j^x)\,,$$

where $D = \frac{A-B}{2}$ and $D' = \frac{A'-B'}{2}$. With the conventions introduced in Fig. 1, the spin Hamiltonian now reads

$$\begin{aligned}
\mathscr{H} \;=\; & \sum_{\mathbf{R}} J\,\mathbf{S}_{\mathbf{R},1}\cdot\mathbf{S}_{\mathbf{R},2} + K\,S^z_{\mathbf{R},1}S^z_{\mathbf{R},2} + D(S^x_{\mathbf{R},1}S^y_{\mathbf{R},2} + S^y_{\mathbf{R},1}S^x_{\mathbf{R},2}) \\
& + \frac{C}{\sqrt{2}}(S^y_{\mathbf{R},1}S^z_{\mathbf{R},2} + S^z_{\mathbf{R},1}S^y_{\mathbf{R},2} - S^x_{\mathbf{R},1}S^z_{\mathbf{R},2} - S^z_{\mathbf{R},1}S^x_{\mathbf{R},2}) \\
& + J'\,\mathbf{S}_{\mathbf{R},1}\cdot\mathbf{S}_{\mathbf{R}-\mathbf{b},2} + K'\,S^y_{\mathbf{R},1}S^y_{\mathbf{R}-\mathbf{b},2} + D'(-S^x_{\mathbf{R},1}S^z_{\mathbf{R}-\mathbf{b},2} - S^z_{\mathbf{R},1}S^x_{\mathbf{R}-\mathbf{b},2}) \\
& + \frac{C'}{\sqrt{2}}(-S^x_{\mathbf{R},1}S^y_{\mathbf{R}-\mathbf{b},2} - S^y_{\mathbf{R},1}S^x_{\mathbf{R}-\mathbf{b},2} - S^z_{\mathbf{R},1}S^y_{\mathbf{R}-\mathbf{b},2} - S^y_{\mathbf{R},1}S^z_{\mathbf{R}-\mathbf{b},2}) \\
& + J'\,\mathbf{S}_{\mathbf{R},1}\cdot\mathbf{S}_{\mathbf{R}+\mathbf{a}-\mathbf{b},2} + K'\,S^x_{\mathbf{R},1}S^x_{\mathbf{R}+\mathbf{a}-\mathbf{b},2} + D'(S^y_{\mathbf{R},1}S^z_{\mathbf{R}+\mathbf{a}-\mathbf{b},2} + S^z_{\mathbf{R},1}S^y_{\mathbf{R}+\mathbf{a}-\mathbf{b},2}) \\
& + \frac{C'}{\sqrt{2}}(-S^x_{\mathbf{R},1}S^y_{\mathbf{R}+\mathbf{a}-\mathbf{b},2} - S^y_{\mathbf{R},1}S^x_{\mathbf{R}+\mathbf{a}-\mathbf{b},2} + S^x_{\mathbf{R},1}S^z_{\mathbf{R}+\mathbf{a}-\mathbf{b},2} + S^z_{\mathbf{R},1}S^x_{\mathbf{R}+\mathbf{a}-\mathbf{b},2}) \\
& + J_2\,\mathbf{S}_{\mathbf{R},1}\cdot(\mathbf{S}_{\mathbf{R}+\mathbf{a},1} + \mathbf{S}_{\mathbf{R}+\mathbf{b},1} + \mathbf{S}_{\mathbf{R}+\mathbf{a}-\mathbf{b},1}) + J_2\,\mathbf{S}_{\mathbf{R},2}\cdot(\mathbf{S}_{\mathbf{R}+\mathbf{a},2} + \mathbf{S}_{\mathbf{R}+\mathbf{b},2} + \mathbf{S}_{\mathbf{R}+\mathbf{a}-\mathbf{b},2}) \\
& + J_3\,\mathbf{S}_{\mathbf{R},1}\cdot(\mathbf{S}_{\mathbf{R}+\mathbf{a},2} + \mathbf{S}_{\mathbf{R}-\mathbf{a},2} + \mathbf{S}_{\mathbf{R}+\mathbf{a}-2\mathbf{b},2})\;, \qquad (10)
\end{aligned}$$

where $\mathbf{R} = n\mathbf{a} + m\mathbf{b}$ labels the unit cells and $j \in \{1,2\}$ labels the sublattice index.

The numerical values of the above coupling parameters, as found by *ab initio* multireference configuration-interaction (MRCI) calculations, are (in units of meV) :

Bond type b	$J_b^{(0)}$	A_b	B_b	C_b	$J_b = J_b^{(0)} + \frac{A_b+B_b}{2}$	$K_b = -\frac{3}{2}(A_b+B_b)$	$\Gamma_{xy}^b = \frac{A-B}{2}$	$\Gamma_{zx}^b = -\Gamma_{yz}^b$
1	−11.2	−0.33	2.2	4.0	−10.2	−2.9	−1.3	2.8
2	−1.5	7.5	0.30	2.2	2.4	−11.7	+3.6	1.6
3	−1.5	7.5	0.30	2.2	2.4	−11.7	+3.6	1.6

For completeness, we also provide the coupling parameters as found by spin-orbit CASSCF calculations (in units of meV) :

Bond type b	$J_b^{(0)}$	A_b	B_b	C_b	$J_b = J_b^{(0)} + \frac{A_b+B_b}{2}$	$K_b = -\frac{3}{2}(A_b+B_b)$	$\Gamma_{xy}^b = \frac{A_b-B_b}{2}$	$\Gamma_{zx}^b = -\Gamma_{yz}^b$
B1($b=1$)	−14.7	−0.6	0.44	−2.3	−14.8	0.24	−0.52	−1.6
B2($b=2$)	−3.5	4.2	−1.2	1.2	−2.2	−4.5	+2.7	0.86
B3($b=3$)	−3.5	4.2	−1.2	1.2	−2.2	−4.5	+2.7	0.86

References

1. Figgen, D., Peterson, K. A., Dolg, M. & Stoll, H. Energy-consistent pseudopotentials and correlation consistent basis sets for the 5d elements Hf–Pt. *J. Chem. Phys.* **130**, 164108 (2009).
2. Dunning, T. H. Gaussian basis sets for use in correlated molecular calculations. i. the atoms boron through neon and hydrogen. *J. Chem. Phys.* **90**, 1007–1023 (1989).
3. Fuentealba, P., Preuss, H., Stoll, H. & von Szentpaly, L. A proper account of core-polarization with pseudopotentials: single valence-electron alkali compounds. *Chem. Phys. Lett.* **89**, 418 (1982).
4. Katukuri, V. M., Stoll, H., van den Brink, J. & Hozoi, L. *Ab initio* determination of excitation energies and magnetic couplings in correlated quasi-two-dimensional iridates. *Phys. Rev. B* **85**, 220402 (2012).
5. Katukuri, V. M. *et al.* Kitaev interactions between $j = 1/2$ moments in honeycomb Na_2IrO_3 are large and ferromagnetic: insights from *ab initio* quantum chemistry calculations. *New J. Phys.* **16**, 013056 (2014).

6. Bogdanov, N. A., Katukuri, V. M., Stoll, H., van den Brink, J. & Hozoi, L. Post-perovskite $CaIrO_3$: A $j = 1/2$ quasi-one-dimensional antiferromagnet. *Phys. Rev. B* **85**, 235147 (2012).
7. Werner, H.-J., Knowles, P. J., Knizia, G., Manby, F. R. & Schütz, M. MOLPRO 2012, see http://www.molpro.net.
8. Fink, K., Fink, R. & Staemmler, V. Ab Initio Calculation of the Magnetic Exchange Coupling in Linear Oxo-Bridged Binuclear Complexes of Titanium(III), Vanadium(III), and Chromium(III). *Inorg. Chem.* **33**, 6219 (1994).
9. van Oosten, A. B., Broer, R. & Nieuwpoort, W. C. Heisenberg exchange enhancement by orbital relaxation in cuprate compounds. *Chem. Phys. Lett.* **257**, 207 (1996).
10. Broer, R., Hozoi, L. & Nieuwpoort, W. C. Nonorthogonal Approaches to the Study of Magnetic Interactions. *Mol. Phys.* **101**, 233 (2003).
11. Calzado, C. J., Evangelisti, S. & Maynau, D. Local Orbitals for the Truncation of Inactive Space: Application to Magnetic Systems. *J. Phys. Chem. A* **107**, 7581 (2003).
12. Katukuri, V. M. *et al.* Mechanism of basal-plane antiferromagnetism in the spin-orbit driven iridate Ba_2IrO_4. *Phys. Rev. X* **4**, 021051 (2014).
13. Bogdanov, N. A., Maurice, R., Rousochatzakis, I., van den Brink, J. & Hozoi, L. Magnetic State of Pyrochlore $Cd_2Os_2O_7$ Emerging from Strong Competition of Ligand Distortions and Longer-Range Crystalline Anisotropy. *Phys. Rev. Lett.* **110**, 127206 (2013).
14. Pipek, J. & Mezey, P. G. A fast intrinsic localization procedure applicable for abinitio and semiempirical linear combination of atomic orbital wave functions. *J. Chem. Phys.* **90**, 4916 (1989).
15. Yildirim, T., Harris, A. B., Aharony, A. & Entin-Wohlman, O. Anisotropic spin Hamiltonians due to spin-orbit and Coulomb exchange interactions. *Phys. Rev. B* **52**, 10239 (1995).
16. Todorova, V. & Jansen, M. Synthesis, Structural Characterization and Physical Properties of a New Member of Ternary Lithium Layered Compounds - Li_2RhO_3. *Z. Anorg. Allg. Chem.* **637**, 37 (2011).
17. Jackeli, G. & Khaliullin, G. Mott Insulators in the Strong Spin-Orbit Coupling Limit: From Heisenberg to a Quantum Compass and Kitaev Models. *Phys. Rev. Lett.* **102**, 017205 (2009).
